# CHF6297: a novel potent and selective p38 MAPK inhibitor with robust anti-inflammatory activity and suitable for inhaled pulmonary administration as dry powder

**DOI:** 10.3389/fphar.2024.1343941

**Published:** 2024-03-14

**Authors:** Cataldo Martucci, Andrew Dennis Allen, Nadia Moretto, Valentina Bagnacani, Alessandro Fioni, Riccardo Patacchini, Maurizio Civelli, Gino Villetti, Fabrizio Facchinetti

**Affiliations:** Corporate Pre-Clinical R&D, Chiesi Farmaceutici S.p.A., Parma, Italy

**Keywords:** inflammation, P38 alpha, chronic obstructive pulmonary disease, cytokines, neutrophilia

## Abstract

Inhibition of p38 mitogen-activated protein kinase (MAPKs) is a potential therapeutic approach for the treatment of acute and chronic pulmonary inflammatory conditions. Here, we report the *in vitro* and *in vivo* characterization of the anti-inflammatory effects of CHF6297, a novel potent and selective p38α inhibitor designed for inhalation delivery as a dry powder formulation. CHF6297 has been proven to inhibit p38α enzymatic activity with sub-nanomolar potency (IC_50_ = 0.14 ± 0.06 nM), with >1,000-fold selectivity against p38γ and p38δ. In human peripheral blood mononuclear cells (PBMCs) stimulated with lipopolysaccharides (LPS), as well as in human bronchial epithelial cells (BEAS2B) stimulated with TNF-α or cigarette smoke extract (CSE), CHF6297 inhibited interleukin (IL)-8 release with low nanomolar potency. CHF6297 administered to rats by using a nose-only inhalation device as a micronized dry powder formulation blended with lactose dose-dependently inhibited the LPS-induced neutrophil influx in the bronchoalveolar lavage fluid (BALF). CHF6297 administered intratracheally to rats dose-dependently counteracted the IL-1β (0.3 mg/kg)-induced neutrophil influx (ED_50_ = 0.22 mg/kg) and increase in IL-6 levels (ED_50_ = 0.82 mg/kg) in the BALF. In mice exposed to tobacco smoke (TS), CHF6297, administered intranasally (i.n.) for 4 days at 0.03 or 0.3 mg/kg, dose-dependently inhibited the corticosteroid-resistant TS-induced neutrophil influx in the BALF. In a murine house dust mite (HDM) model of asthma exacerbated by influenza virus A (IAV) (H3N3), CHF6297 (0.1 mg/kg, i.n.) significantly decreased airway neutrophilia compared to vehicle-treated IAV/HDM-challenged mice. When CHF6297, at a dose ineffective *per se* (0.03 mg/kg), was added to budesonide, it augmented the anti-inflammatory effects of the steroid. Overall, CHF6297 effectively counteracted lung inflammation in experimental models where corticosteroids exhibit limited anti-inflammatory activity, suggesting a potential for the treatment of acute exacerbations associated with chronic obstructive pulmonary disease (COPD) and asthma, acute lung injury (ALI), and viral-induced hyperinflammation.

## Introduction

The p38 mitogen-activated protein kinase (MAPK) signaling pathway plays an essential role in inflammation, especially in the regulation and activation of key proinflammatory mediators. There are four known human isoforms of p38 MAPK, namely, p38α, (MAPK14) p38β, (MAPK11) p38γ (MAPK12), and p38δ (MAPK13), which differ in cell and tissue distribution, regulation of kinase activation, and phosphorylation of downstream substrates (Hale et al., 1999; Dominguez et al., 2005). These enzymes also differ in sensitivity to p38 MAPK inhibitors (Dominguez et al., 2005). The p38 kinases are activated by physical and chemical stress factors (e.g., UV, cigarette smoke, aeroallergens, and airborne pollutants), proinflammatory cytokines, and viral and bacterial pathogens (Haddad et al., 2001) and are responsible for phosphorylating and activating transcription factors (such as ATF-2, MAX, CHOP, and C/ERPb) as well as other kinases (such as MAPKAP-K2/3 or MK2/3) (Cuenda and Rousseau, 2007). The most thoroughly studied is the p38α isoform, which represents a point of convergence for multiple signaling processes that are activated during inflammation and plays a central role in the regulation and activation of key proinflammatory mediators (Dominguez et al., 2005). In the lungs of chronic obstructive pulmonary disease (COPD) patients, the expression of activated p38α is increased in comparison with healthy controls and correlated with the degree of lung function impairment and alveolar wall inflammation (Gaffey et al., 2013). p38α inhibitors showed anti-inflammatory effects in a range of animal models of airway inflammation and reduced cytokine production from COPD alveolar macrophages, lung lymphocytes, and bronchial epithelial cells *in vitro* (Chung, 2011). Moreover, p38α inhibition may be beneficial in COPD and corticosteroid-insensitive asthma because of restoring corticosteroid sensitivity (Chung, 2011). Orally administered inhibitors such as losmapimod (Pascoe et al., 2017), dilmapimod (SB-681323) (Singh et al., 2010), and PH-797804 (Singh et al., 2015) have been profiled for a range of indications including COPD, rheumatoid arthritis, pain, and atherosclerosis (Norman, 2015). Until now, chronic treatments with oral p38α inhibitors did not convincingly deliver significant clinical benefits in COPD patients. However, the oral p38α inhibitor BCT-197 (acumapimod) may still hold promise as a therapy for acute exacerbations of COPD (Strâmbu et al., 2019). Liabilities for oral p38 inhibitors have been associated with dose-limiting systemic toxicities such as increased liver enzymes and rash (Pascoe et al., 2017). Novel p38α inhibitors that need to be administered directly into the lung by inhalation have been developed with the aim of limiting systemic exposure and the associated side effects. Pfizer identified PF-03715455, which has undergone clinical evaluation for COPD and asthma (Millan et al., 2011). AstraZeneca developed AZD7624, an inhaled p38α inhibitor that failed to show any benefit in patients with COPD when tested in a phase 2 clinical study (Patel et al., 2018). Here, we report the characterization of CHF6297, previously coded as compound 4e (Armani et al., 2022), a highly potent and selective p38α inhibitor endowed with robust *in vitro* and *in vivo* anti-inflammatory properties and suitable for inhaled delivery as dry powder.

## Materials and methods

### Animals

Adult male Wistar rats (250–350 gr; Charles River, Calco, Italy) were housed and managed according to a set of principles and a series of standard operating procedures following the latest and most comprehensive international and national guidelines (ETS no. 123, Directive 2010/63/EU, Recommendation 526/2007 EC, Dlgs 26/2014 Italian transposition of the Directive). Experimental protocols and procedures were approved by the Chiesi Farmaceutici S.p.A. Ethical Committee.

### Chemicals

**FIGURE 1:**
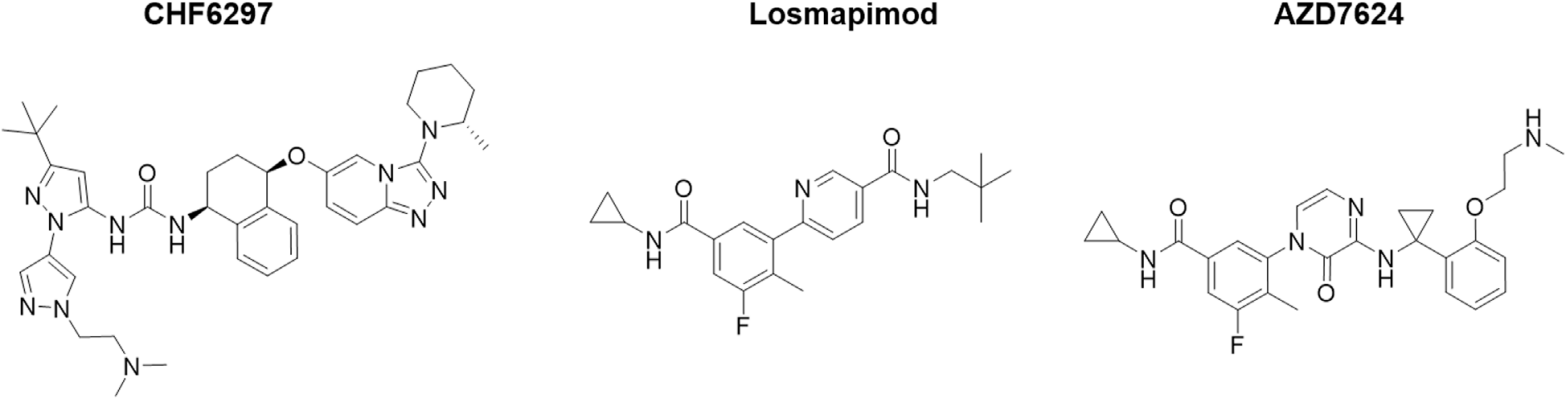
Chemical structures of the p38α inhibitors CHF6297, losmapimod, and AZD7624.

The chemical structures of CHF6297, AZD7624, and losmapimod are shown in Figure 1, and they were synthesized by Chiesi Farmaceutici S.p.A. (Parma, Italy). Budesonide and lipopolysaccharides (LPSs) from *Escherichia coli* 0111:B4 (Code #L3012) were purchased from Sigma-Aldrich. Recombinant interleukin (IL)-1β (Code # 400-01B) was purchased from PeproTech.

### Enzymatic assays

The inhibitory activity of CHF6297 was determined using an AlphaScreen^®^ (PerkinElmer)-based kinase activity assay as previously described (Armani et al., 2022). Kinase reactions consisted of 25 mM 4-(2-hydroxyethyl)-1-piperazine ethanesulfonic acid (HEPES), pH 7.5; 10 mM MgCl_2_; 100 μM Na_3_VO_4_; 2 mM DTT; 0.05 mg/mL Tween^TM^ 20; 100 pM solution of p38α, p38β, p38δ, or p38γ (Invitrogen); 1% DMSO; and 0.3 μg/mL ATF-2 fusion protein (New England Biolabs). Compounds were incubated under these conditions for 2 h, at 25°C, prior to the initiation of kinase activity by the addition of 250 μM ATP. After 1 h at 25°C, the reactions were stopped by adding 25 mM HEPES pH 7.5 containing 62.5 mM ethylenediaminetetraacetic acid (EDTA), 0.05% Triton X-100, 10% BSA, and 0.83 ng/μL anti-phospho-ATF-2 antibody (Abcam, ab28812). Detection was performed by measuring luminescence following the addition of AlphaScreen donor beads (PerkinElmer, 6765300) and Protein A AlphaScreen acceptor beads (PerkinElmer, 6760137), both at a final concentration of 20 μg/mL. IC_50_ values were determined from concentration–response curves. Compounds were tested in at least three independent experiments, unless stated otherwise.

### TNF-α release in LPS-stimulated PBMCs

Peripheral blood mononuclear cells (PBMCs) were isolated from the blood of healthy human volunteers and Wistar rats (250–350 gr; Charles River, Calco, Italy) using a standard density gradient centrifugation technique. The citrated blood was placed onto Histopaque^TM^ and centrifuged. The PBMCs were then removed from the density gradient interface and washed in phosphate-buffered saline (PBS). The PBMCs were suspended in RPMI 1640 medium (without serum), aliquoted into a 96-well plate, and incubated at 37°C for 3 h in a humidified incubator. After incubation, the medium was replaced (with a medium containing 1% fetal bovine serum), and the plate was incubated at 37°C, for 1 h, in the presence of a test compound or the appropriate vehicle. LPS (10 ng/mL), or an appropriate vehicle control, was then added to the cells, and the plate was returned to the incubator for 18 h. Cell-free supernatants were removed and assayed for TNF-α levels using MSD plates on the SECTOR Imager 6000 (Meso Scale).

### CSE- and TNF-α evoked IL-8 release in normal human bronchial smooth muscle cells

Cigarette smoke extract (CSE) was generated as reported previously (Moretto et al., 2012). Briefly, aqueous CSE was obtained from the combustion of four cigarettes (Marlboro Red, 12 mg tar, 0.9 mg nicotine each) bubbled through 50 mL of the culture medium and subsequently filtered through a 0.2-μm-pore filter (Millipore, Billerica, MA). To ensure reproducibility among different CSE batches, the absorbance [optical density (OD)] measured at 320 nm was used as a measure of the “strength” of the extract. Dilutions were made with culture media to obtain the desired absorbance. The CSE was freshly prepared on the day of the experiment and immediately used after preparation. Human bronchial smooth muscle cells (HBSMCs) were seeded in DMEM with 10% FBS (50 U/mL penicillin, 0.05 mg/mL streptomycin, and 2 mM L-glutamine) in 48-well plates at a density of 10,000 cells/well, grown to ∼80%–90% confluence and incubated overnight in serum-free DMEM before treatments. All the treatments were performed in serum-free DMEM. The cells were pretreated for 30 min with either the vehicle or CHF6297 or losmapimod before stimulation with freshly prepared CSE, TNF-α, (1 ng/mL), or the vehicle for 18 h at 37°C. Human IL-8 was measured in the collected supernatants by use of a paired-antibody quantitative ELISA kit (BioSource International, Camarillo, CA).

### Inhalation pharmacokinetics in rats

Inhalation pharmacokinetics (PK) of CHF6297 as micronised dry powder blended with lactose 20% w/w was investigated in male Wistar rats at 2 dose levels (0.1 and 1 mg/kg) using the nose-only inhalation delivery system (Figure 8). For each PK study, 21 rats were placed in suitable plastic restrainers and connected to the inhalation tower. Inhalation system parameters were set to achieve a nominal dose of 0.1 mg/kg and 1 mg/kg in 30 min of exposure. The particle size and actual dose are given in Supplementary Material (Table 2). At the end of the inhalation period, the animals were returned to their cages. At the designed timepoint, three animals for each timepoint were anesthetized with sevoflurane and euthanized by bleeding from the abdominal aorta. Then, lung samples were immediately excised, externally washed with cold saline, accurately weighed, homogenized using a temperature-controlled Precellys homogenizer (Bertin Technologies, France), and stored at −80 °C until analysis.

Plasma and lung samples were analyzed by using an HPLC-MS/MS system, consisting of a 4000 QTRAP mass spectrometer (AB Sciex LLC, Framingham, MA, United States) with an electrospray ionization (ESI) source, coupled to an Agilent 1100 HPLC (Agilent Technologies, Santa Clara, CA, United States). Analyte separation was performed using a Gemini C18 column (Phenomenex Inc., Torrance, CA, United States) and water with 0.1% v/v AcOH in the gradient with acetonitrile with 0.1% v/v AcOH as eluents. Sample preparation was performed by protein precipitation using preassembled filtration devices (Mini-UniPrep Syringeless Filters, 0.45 µm PTFE, Whatman, GE Healthcare, Buckinghamshire, United Kingdom). Aliquots of 50 μL of the sample (plasma or homogenate) were transferred to the bottom part of the device, spiked with 10 μL of the appropriate internal standard solution, and 150 μL of acetonitrile was added. The calibration and quality control samples were prepared by adding 10 µL of the CHF6297 working standard solution in acetonitrile to 50 µL of the blank matrix. After vortex mixing, the obtained mixture was filtered by manual compression of the device, and the filtered solution was diluted with 100 μL of water. The Mini-UniPrep devices were directly placed in the autosampler rack of the HPLC system. Pharmacokinetic evaluation and parameter determination (Cmax, Tmax, AUClast, and t_1/2_) were performed with Phoenix 64 software (Certara, New Jersey, United States) using non-compartmental analysis. Since the actual dose was within ±5% of the nominal one, the nominal dose was used for PK calculation.

### LPS/IL-1β i.t. administration

For intratracheal (i.t.) administration of LPS or IL-1β, animals were anesthetized with sevoflurane (4% in oxygen), and a laryngoscope was moved forward into the mouth to visualize the trachea and guide the insertion of a fine-tipped PE 100 tube (air-jet delivery system) directly into the trachea and located 1–2 mm above the carina bifurcation. LPS or IL-1β at a concentration of 5 μg/kg and 0.3 μg/kg, respectively, was prepared as a saline solution in a 0.5 mL/kg volume and was blown into the airways during the spontaneous inspiration phase in an air volume of 2 mL.

### LPS-induced neutrophilia in rats: CHF6297 potency and duration of action

CHF6297 (0.03, 0.1, and 1 mg/kg), prepared as a micronized dry powder blended with lactose and magnesium stearate 2%, was administered by inhalation through a nose-only (n.o.) delivery system: the inhalation system comprised a nose-only inhalation exposure tower, restraining tubes, a powder generator, an earth lead, dried and filtered air supply, and an extract line with a filtration system incorporated in it. Each dose group was allocated to a separate system, and each system was housed in an individual ventilated hood.

Powder generation: Compound and vehicle blends were compressed into canisters by using a hydraulic press at 50 bar to prepare a stable “tablet” and ensure a uniform pack density. The Wright Dust Feeder (WDF) comprised an electric motor, gears, a scraper blade, air entry, and a final jet and baffle array. The scraper blade scraped off a fine layer of powder, driven by the motor, and the output speed was determined by the configuration of the gears. The scraped powder was then blown out of the dust feed at its base through a jet which focused the powder stream at a baffle, thus reducing aggregation and producing a fine particulate. The WDF was attached to an aerosol conditioning pre-chamber for the delivery of the powder to the inhalation tower. The generation of the powder was monitored throughout the exposure periods either visually or electronically with a dust monitor.

Inhalation tower: The nose-only inhalation tower was a modular apparatus of anodized aluminum comprising a base unit, two animal sections, and a top section, connected to an earth lead to prevent the formation of any static build-up. During the exposures, the rats were held in restraining tubes, with their snouts protruding into the exposure chamber (see Supplementary Material, Figure 1). The rate of airflow through the WDF was 19 L/min, and that of the exhaust airflow was 20 L/min (vehicle, 0.1 and 1 mg/kg doses) or 40 L/min (0.03 mg/kg doses). The air supply and extract were monitored using in-line flowmeters and recorded manually.

The inhaled dose was derived using the following equation (Alexander et al., 2008): delivered dose (μg/kg) = (Conc. × RMV × D)/BW, where Conc. = concentration of CHF-6297 in the air inhaled (µg/L); RMV = volume of air inhaled in 1 min (L/min) 0.608 × BW (kg) 0.852; D = duration of exposure to the aerosol (min); and BW = body weight (kg).

Animals were dosed either 1 h (dose–response curve) or 24 h (duration of action) before LPS challenge. Four hours after endotoxin challenge or saline injection, bronchoalveolar lavage fluid (BALF) was collected for cell counting.

### IL-1β induced neutrophilia and IL-6 accumulation in BALF of rats: CHF6297 potency and duration of action

IL-1β is a potent proinflammatory agent that markedly induces lung neutrophilia when administered intratracheally in rats, and p38α MAPK is involved in IL-1β signaling in lung cells (Laporte et al., 2000). The dose–response curve and anti-inflammatory duration of action studies were carried out by delivering CHF6297 intratracheally through the DP-4M PennCentury insufflator as micronized dry powder (blended with lactose) at the doses of 0.1, 0.35, and 1 mg/kg, 1 h or 24 h before IL-1β challenge. Four hours after IL-1β challenge, BALF was collected for cell counting and IL-6 quantification.

### BALF procedure

Animals were anesthetized with sevoflurane (4% in oxygen) and euthanized by bleeding from the abdominal aorta. BALF was obtained by gently washing the lungs with three aliquots (4 mL each) of 100 mL of solution A [Hank’s balanced salt solution (HBSS) × 10; 100 mL of 100 mM EDTA; 10 mL of 1 mM HEPES; and 790 mL of distilled water]. Routine recovery of the BALF did not significantly differ between animals with ∼ 80% of the instilled volume recovered (9.5–10.5 mL). The resulting BALF was centrifuged at 800 × *g* for 10 min at 4°C. The pellets derived from the same animal were combined and resuspended in a volume of 1 mL, and total and differential cell count assays were performed within 2 h using an automated cell counter (Dasit, Sysmex). The cell count per animal was calculated from the number of cells for 1 µL of BALF multiplied by the volume used for the resuspension of the cell pellet.

For the experimental studies performed with IL-1β challenge, the first aliquot of BALF was centrifuged separately: the resulting supernatant was frozen at −80°C for IL-6 ELISA analysis.

### IL-6 and TNF-α ELISA

Frozen cell-free BALFs were analyzed using an ELISA kit purchased from R&D Systems (Rat IL-6 ELISA Kit; Cat. No. SR6000B, Lot. 307537), according to the manufacturer’s instructions.

The TNF-α level in plasma samples was evaluated using an ELISA kit purchased from Abcam (ab100785), according to the manufacturer’s instructions.

### Neutrophilia induced by cigarette smoke exposure in mice

C57BL6J female mice aged 9–10 weeks (Harlan Laboratories S.r.l) in groups of 8 were exposed, via whole-body inhalation, twice daily 1 h apart, for 4 consecutive days, to the tobacco smoke (TS) (generated using “Marlboro Gold: 9 mg of tar and 0.7 mg nicotine”) of two cigarettes/exposure on days 1 and 2, three cigarettes/exposure on day 3, and four cigarettes/exposure on the last exposure day in clear polycarbonate chambers (27 cm × 16 cm × 12 cm). The smoke was introduced into the exposure chambers (21100-790 Ugo Basile, Biological Research Instruments, Comerio, Varese, Italy), with the airflow generated by using a mechanical ventilator (7025 Rodent Ventilator, Ugo Basile). Each exposure chamber was connected to a different ventilator set at a rate of 100 mL/min (frequency: 50 strokes/min; volume: 2 mL/stroke). At regular intervals (after burning two cigarettes), the exposure chambers were opened and aired. Sham and control mice were exposed to air only in the same manner for the same duration of time.

CHF6297 (0.03 and 0.3 mg/kg) and budesonide (0.3 mg/kg), our reference compound and vehicle (0.2% Tween 80 in saline), respectively, were administered intranasally in a volume of 30 µL, 1 h before and 5 h after TS exposure under light isoflurane anesthesia. Both compounds exhibited a sustained lung pharmacokinetic profile in mice after the intranasal route of administration (data not shown).

A BAL was performed using a volume of 0.6 mL of phosphate-buffered saline that was gently instilled and withdrawn three times using a 12-mL syringe. BALF cells were separated by centrifugation (10 min at 3,070 g), and the supernatant was removed. The resulting cell pellet was resuspended in a known volume of PBS, and the total cell numbers were calculated by counting a stained (Turk’s stain) aliquot under a microscope using a hemacytometer. The cell pellet was resuspended to approximately 10^5^ cells per ml, and cytospin slides were prepared by centrifugation (8 min at 72.26 g; Shandon Cytospin; Thermo Shandon Ltd., Runcorn, Cheshire, United Kingdom). The slides were air-dried and stained using Wright–Giemsa stain, following the manufacturer’s instructions. A differential cell count was performed using light microscopy. Approximately 400 cells were counted from each slide. The cells were identified using standard morphometric techniques.

### Influenza virus A × 31 (H3N2) induced exacerbation of HDM-sensitized Balb/C mice

A mouse model of allergen-driven pulmonary inflammation exacerbated by influenza virus A (IAV) challenge was used (Wang et al., 2021). Male Balb/c 20–25 g mice purchased from Charles Rivers Ltd. (United Kingdom) were used for this study. House dust mite (HDM) extract was obtained from Greer Labs (Lenoir, NC) as a lyophilized preparation of milled mites (*Dermatophagoides pteronyssinus*). Immunization was conducted with HDM at 50 µg/mouse administered subcutaneously with Freund’s complete adjuvant (FCA) on day 0 and day 7. On day 14, the mice received 10 µg of HDM given intranasally under isoflurane anesthesia. The house dust mite extract was instilled into each nostril in a drop-wise fashion, alternating between the two until a volume of 40 µL was delivered. IAV ×31 (H3N2) was grown in Madin-Darby Canine Kidney cells (MDCKs) (European Collection of Cell Cultures). The virus was obtained from the American Type Culture Collection and passaged five times in MDCK cells before purification. The virus was then titrated on MDCK cells by use of standard methods and inactivated by exposure to UV light at 1,200 mJ/cm^2^ for 30 min. On day 15, the mice were infected intranasally under isoflurane anesthesia. The H3N2 or UV-inactivated IAV was instilled into each nostril in a drop-wise fashion, alternating between the two until a volume of 50 µL had been delivered. Budesonide at 0.1 mg/kg (air jet delivery system) and CHF6297 at 0.03 and 0.1 mg/kg or the combination of budesonide and CHF6297 (i.n., 15 µL per nostril, 30 µL total) were administered twice daily, 6 h apart, for 3 consecutive days starting from day 15, 1 h before IAV challenge, to day 17 before BALF collection. The vehicle for both budesonide and CHF6297 was 0.2% Tween 80 in saline. Then, 72 h post viral challenge, the animals were euthanized by cervical dislocation, and the BALF was obtained as described previously. The total and differential cell counts of the BAL fluid samples were measured using an XT-2000iV analyzer (Sysmex).

### Intravenous LPS induced TNF-α release in the rat blood model

Rats were lightly anesthetized and given vehicle (0.2% Tween 80 in saline) or compounds, i.t., using compressed air through an air jet apparatus and a dosing needle. Five minutes prior to the LPS challenge, the rats were placed in a heating chamber (37°C–40°C) to encourage vasodilation. Two hours after compound/vehicle administration, the rats were removed from the heating chamber and placed in a restraining tube. LPS was administered into the lateral tail vein by using a syringe and needle. Five minutes prior to tail vein bleeding, the rats were placed in a heating chamber (37°C–40°C) to encourage vasodilatation. One hour after LPS dosing, lateral tail vein bleeds were carried out. Blood was centrifuged (3,000 × g for 10 min), and plasma was collected and stored at −80°C. TNF-α levels were quantified by ELISA.

### Data analysis

All data are presented as mean ± SEM. Statistical analysis was performed on raw data using one-way analysis of variance (ANOVA), followed by Dunnett’s *post hoc* test for comparisons by using GraphPad software, version 6.0. *p* < 0.05 and *p* < 0.01 were considered the levels of statistical significance.

## Results

### *In vitro* pharmacological characterization of CHF6297

CHF6297 was tested in biochemical enzymatic assays to determine its potency as an inhibitor of human p38α (MAPK14) and of the other human p38 isoforms, p38β (MAPK11), p38γ (MAPK13), and p38δ (MAPK12) (Armani et al., 2022). Activity in the primary screening assay was determined using AlphaScreen technology, in which a peptide (ATF2) is phosphorylated by the appropriate GST-tagged p38 protein isoform in the presence of CHF6297 at concentrations from semi-logarithmic dilutions. An assay for rat p38α was also used, with the rat being the main species used for pre-clinical pharmacodynamic and toxicological studies. A summary of CHF6297 inhibitory potencies against rat p38α and the four human p38 enzyme isoforms is given in Table 1.

**TABLE 1:** The data (*n* = 3) summarized in the table show CHF6297 enzymatic inhibitory potency against 4 human p38 isoforms, namely, p38α, p38β, p38γ, and p38δ, and rat p38α. Notably, IC_50_ values are comparable between human and rat p38α. Kd values for CHF6297 were determined through a KINOMEscan competition binding assay at DiscoverX against human p38 isoforms.

Species	Isoform	Kd (mean ± s.d.)	IC_50_ (nM) (mean ± s.d.)	IC_50_ fold-difference vs. human p38**α**
Human	p38**α**	**0.24 ± 0.06**	**0.14 ± 0.06**	**1**
Human	p38**β**	**1.11 ± 0.6**	**0.20 ± 0.017**	**1.4**
Human	p38**γ**	**>1,000**	**945 ± 58**	**6,750**
Human	p38**δ**	**>1,000**	**>1,000**	**>7,000**
Rat	p38**α**	**n.d.**	**0.5 ± 0.18**	**3.5**

Half maximal inhibitory concentration (IC50), Equilibrium dissociation constant (KD).

The Kd binding values of CHF6297 against the human p38α and p38β enzyme isoforms are reported in Table 1. The KINOMEscan^®^ platform (DiscoverX) was used, which uses a novel and proprietary active site-directed competition binding assay to quantitatively measure the interactions between human p38 isoforms and the target compound.

**FIGURE 2:**
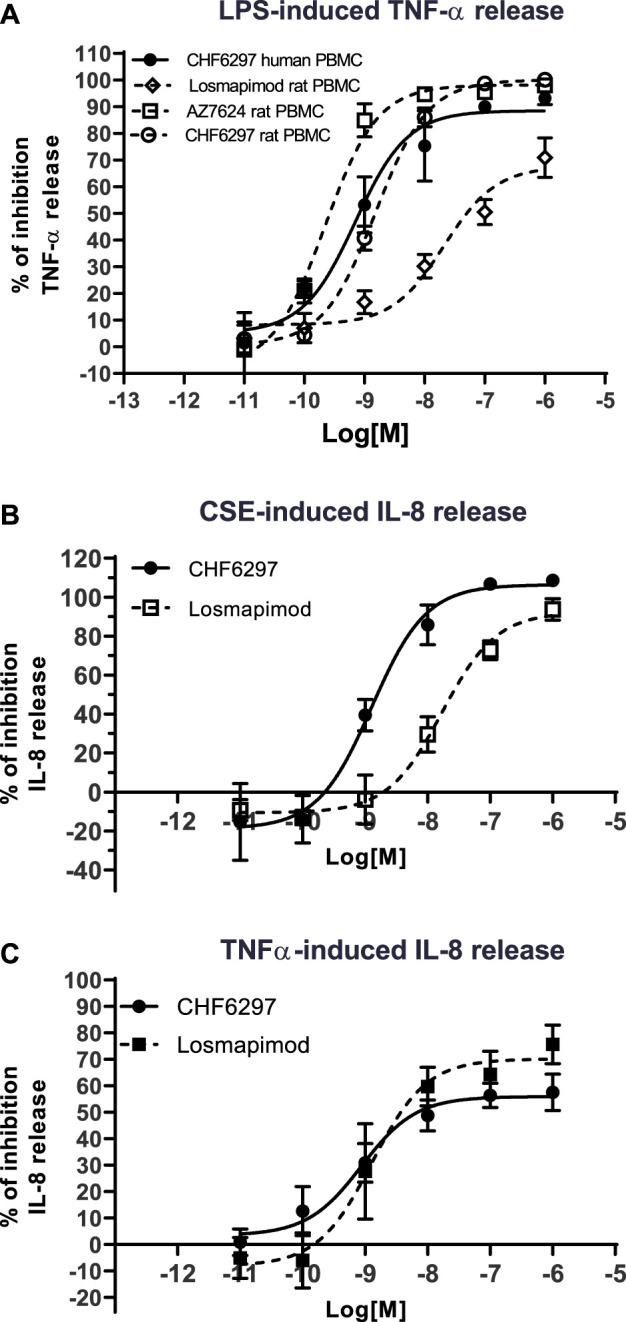
Inhibitory concentration–response curves of CHF6297, AZD7624, and losmapimod on TNF-α release induced by lipopolysaccharides (LPS) in rat and human peripheral blood mononuclear cells (PBMCs) **(A)**, on cigarette smoke extract (CSE)-evoked IL-8 release in normal human bronchial smooth muscle cells **(B)** and IL-8 release induced by TNF-α in BEAS-2B **(C)**. Each point represents the mean ± SD of *n* = 3 independent experiments performed in triplicate.

In rat PBMCs (Figure 2), all compounds displayed potencies in the low nanomolar range: CHF6297, IC_50_ = 1.4 nM (c.i. 1.2–1.6); AZD7624, IC_50_ = 0.22 nM (c.i. 0.1–0.4); and losmapimod, IC_50_ = 11.9 nM (c.i. 3.1–46.3). Thus, in this assay, CHF6297 was ∼11 times more potent than losmapimod and 7-fold less potent than AZD7624. Furthermore, CHF6297 was tested against IL-8 release evoked by CSE in HBSMCs (Moretto et al., 2009). In this assay, both CHF6297 and losmapimod inhibited IL-8 up to a maximum of 100% with the following potencies: CHF6297, IC_50_ = 0.79 nM (c.i. 0.30–2.09) and losmapimod, IC_50_ = 9.13 nM (c.i. 0.9–96.4) (Figure 2). Finally, CHF6297 was tested against IL-8 release evoked by TNF-α, showing a comparable potency.

### Inhaled dose of lactose-blended dry powder of CHF6297

Measured aerosol concentrations of CHF6297 lactose-blended dry powder administered to the experimental animals using the snout-only tower (Figure 5) resulted in an estimated inhaled dose close to the target at all three doses used and showed good inter-experiment reproducibility (Supplementary Tables S3–S5).

### LPS induced BALF neutrophilia in rats: potency and duration of action

**FIGURE 3:**
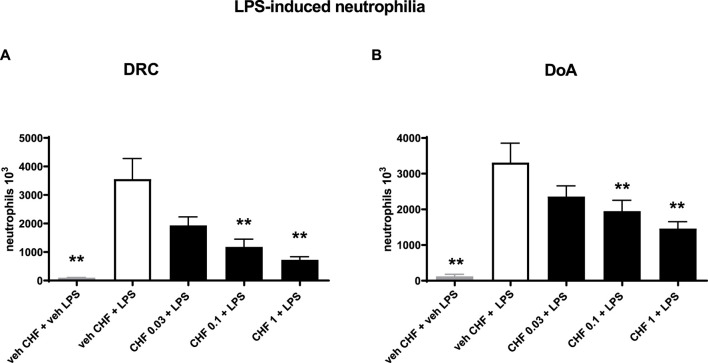
Inhibition of neutrophil count in bronchoalveolar lavage fluid (BALF) in endotoxin (LPS)-exposed rats treated with CHF6297. CHF6297 (CHF 0.03, 0.1, and 1 mg/kg) was administered using the nose-only delivery system as micronized dry powder (in a lactose vehicle) 1 h, panel on the left **(A)**, or 24 h before, panel on the right **(B)**, the LPS challenge. BALF was collected 4 h after the challenge. Neutrophil counts were performed. Bars are the counts of neutrophils in BALF. Treatment groups were compared with the LPS/vehicle-treated group by using analysis of variance, followed by Dunnett’s test. *p < 0.05 and **p < 0.01 for treatment groups compared with the vehicle + LPS control group. Values are expressed as the mean ± SEM values of each treatment group (*n* = 10).

CHF6297 (0.03, 0.1, and 1 mg/kg) was administered using a nose-only (n.o.) delivery system, 1 h, and 24 h before LPS challenges, respectively. BALF was collected for cell counting 4 h after endotoxin administration. When administered 1 h before the inflammatory stimulus, CHF6297 induced a dose-dependent inhibition of pulmonary neutrophilia induced by endotoxin challenge, which reached statistical significance at 0.1 mg/kg (1.18 × 10^6^ ± 0.27 × 10^6^ neutrophils, **p < 0.01) and was maximal at 1 mg/kg (0.7 × 10^6^ ± 0.12 × 10^6^ neutrophils, **p < 0.001) compared to the LPS challenge-treated group (3.56 × 10^6^ ± 0.72 × 10^6^ neutrophils). The calculated ED_50_ value was 0.064 mg/kg, (CI: 0.02–0.16) (Figure 3A). When CHF6297 was administered 24 h before the LPS challenge, using the n.o. delivery apparatus for 30 min, its inhibitory effect to prevent neutrophil influx in the BALF, induced by LPS challenge, was still maintained, starting from a dose of 0.1 mg/kg (1.9 × 10^6^ ± 0.3 × 10^6^ neutrophils, *p < 0.05) and reaching the maximum effect at a dose of 1 mg/kg (1.45 × 10^6^ ± 0.19 × 10^6^ neutrophils, **p < 0.01) compared to the LPS challenged rats (3.3 × 10^6^ ± 0.54 × 10^6^ neutrophils) (Figure 3B).

### IL-1β induced BALF neutrophilia in rats: potency and duration of action

**FIGURE 4:**
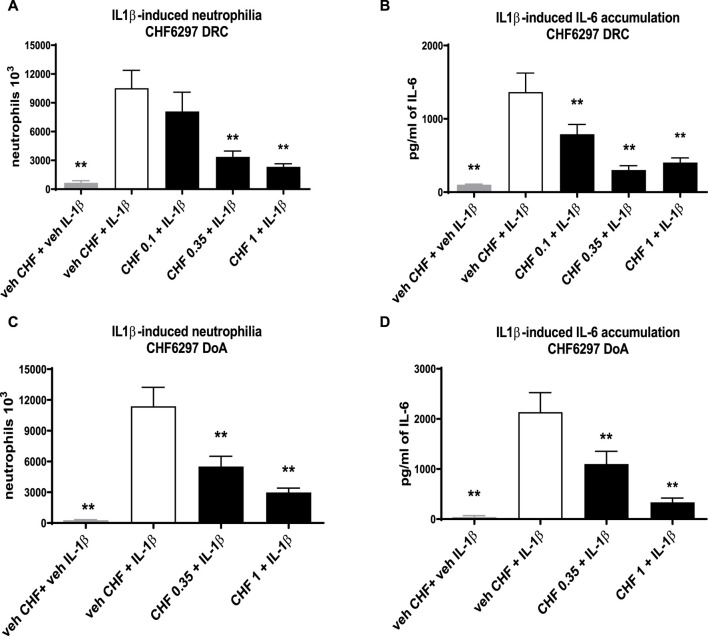
Inhibition of the neutrophil recruitment and interleukin (IL)-6 accumulation in the bronchoalveolar lavage fluid in interleukin (IL)-1β-exposed rats treated with CHF6297. CHF6297 (CHF 0.1, 0.35, and 1 mg/kg) was administered using the DP-4M PennCentury insufflator as a micronized dry powder (in the lactose vehicle) 1 h, panels **(A, B)**, or 24 h before, panels **(C, D)**, the IL-1β challenge. BALF was collected 4 h after the challenge. Neutrophil counts and IL-6 quantification were performed. Bars are the count of neutrophils [panels **(A, C)**] and IL-6 concentration [panels **(B, D)**]. Treatment groups were compared with the IL-1β-/vehicle-treated group by using analysis of variance, followed by Dunnett’s test. *p < 0.05 and **p < 0.01 for the treatment groups compared with the vehicle + IL-1β control group. Values are expressed as the mean ± SEM values of each treatment (*n* = 8).

The data given in Figure 4A clearly show that CHF6297 was efficacious in a dose–response manner when dosed i.t. as a dry powder at 0.35 mg/kg (3.4 × 10^6^ ± 498 neutrophils, **p < 0.01) or 1 mg/kg (2.3 × 10^6^ ± 234 neutrophils, **p < 0.01) compared to the IL-1β challenge group (10.5 × 10^6^ ± 1,853 neutrophils). The estimated anti-inflammatory potency is ED_50_ = 0.22 mg/kg (CI: 0.08–0.375). The IL-6 concentration in the BAL fluid samples collected from this experiment also displayed a significant dose-dependent inhibition at all doses tested (0.1 mg/kg: 791 ± 131 pg/mL, **p < 0.01; 0.35 mg/kg: 302 ± 58.34, **p < 0.01; 1 mg/kg: 404 ± 108, **p < 0.01; IL-1β: 1,366 ± 258), tracking the effect observed on neutrophils (Figure 4B). When the pre-dose time was extended to 24 h, CHF6297 continued to display efficacy, as shown by the data in Figure 4C, where both doses tested, 0.35 (5.5 × 10^6^ ± 814 neutrophils, **p < 0.01) and 1 mg/kg (2.98 × 10^6^ ± 62 neutrophils, **p < 0.01), showed significant activity when compared to the challenge group (11.4 × 10^6^ ± 1,911 neutrophils). This is confirmed by the measured concentration of IL-6 shown in Figure 4D for the same experiment: CHF6297 at both the doses tested, 0.35 mg/kg (1,101 ± 253 pg/mL, **p < 0.01) and 1 mg/kg (337 ± 84 pg/mL, **p < 0.01), significantly reduced the induced increase in IL-6 (2,137 ± 387 pg/mL) concentration.

### BALF neutrophilia induced by acute TS exposure in mice

**FIGURE 5:**
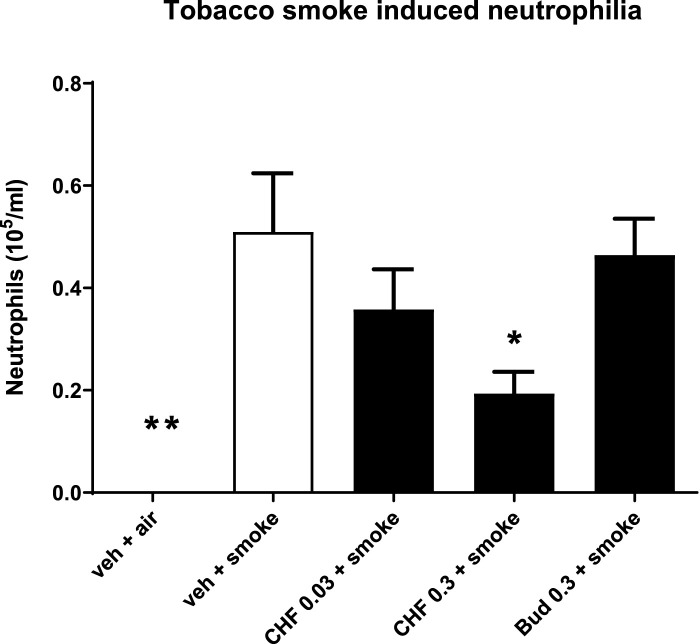
Effect of CHF6297 on neutrophilia induced by tobacco smoke (TS) exposure in mice. CHF6297 (0.03–0.3 mg/kg) or vehicle was administered intranasally to lightly anesthetized mice twice a day 1 h before and 5 h after TS exposure. BALF was collected 24 h after the last TS exposure, and differential cell counts were performed. Values are expressed as the mean ± SEM (*n* = 9–10) of the number of neutrophils recovered in BALF. In vehicle-treated animals exposed to air, neutrophils in BALF were undetectable. Statistical analysis was performed by one-way analysis of variance, followed by Bonferroni correction for multiple comparisons between treatment groups. *p < 0.05 and **p < 0.01 for treatment groups compared with the vehicle + TS control group.

Mice treated intranasally with vehicle and exposed to TS showed significant lung neutrophilia compared with the group of mice exposed to air (air: 0.0 ± 0.0 × 10^5^ cells/mL; smoke: 0.5 × 10^5^ cells/mL; **p < 0.01). Intranasal treatment with doses of 0.03 and 0.3 mg/kg of CHF6297 every day 1 h before and 5 h after TS exposure produced a dose-dependent inhibition of neutrophilia in the BALF, which reached statistical significance at the highest dose (smoke: 0.5 ± 0.1 × 10^5^ cells/mL, 0.3 mg/mL CHF: 0.2 ± 0.1 × 10^5^, % inhibition: 62.0 ± 8.4, *p < 0.05). The lower dose provided a modest and not statistically significant inhibition of neutrophil recruitment (0.03 mg/kg CHF6297: 0.4 ± 0.1 × 10^5^ cells/mL, *p* > 0.05, % of inhibition: 29.7 ± 15.3). Intranasal treatment of mice with a dose of 0.3 mg/kg of budesonide every day 1 h before TS exposure did not alter TS-induced neutrophilia (smoke: 0.5 ± 0.1 × 10^5^ cells/mL; 0.3 mg/kg budesonide: 0.5 ± 0.1 × 10^5^ cells/mL, *p* > 0.05) Figure 5.

### Effects of CHF6297 on IAV-induced acute exacerbation in a mouse model of HDM-driven allergic pulmonary inflammation

**FIGURE 6:**
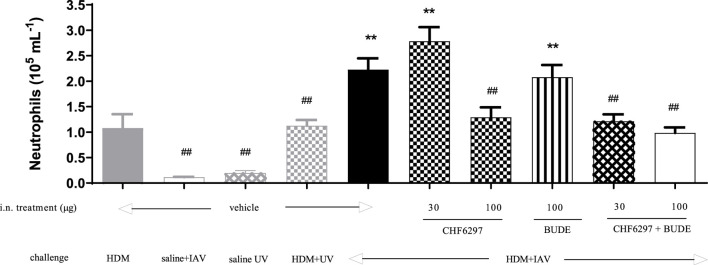
Effect of CHF6297 on influenza A virus (IAV)-induced neutrophilic exacerbation in house dust mite (HDM)-challenged mice. Each bar represents the mean ± SEM. Treatment legends are self-explicative and appear at the *X*-axis, with “UV” referring to inactivated IAV. Groups treated with the test compounds were compared to HDM + UV + Veh (**) and HDM + IAV + Veh (##) by using one-way ANOVA, followed by Dunnett’s *t*-test.

CHF6297 was tested as a single i.n. treatment at the doses of 30 and 100 μg/kg and in combination with budesonide (100 μg/kg i.n.) in a virus-dependent murine model of severe asthma (Wang et al., 2021). As shown in Figure 6, the control group treated with vehicle and exposed to HDM + UV (inactivated virus) showed an increased number of neutrophils in the BAL fluid (1.12 ± 0.11 × 10^5^ cells/mL), and the extent of the effect was superimposable to the that observed in the group treated with HDM alone (1.08 ± 0.26 × 10^5^ cells/mL). The challenge with IAV significantly improved neutrophil recruitment, simulating an exacerbation event (2.23 ± 0.22 × 10^5^ cells/mL, HDM + UV = 1.12 ± 0.11 × 10^5^ cells/mL, **p < 0.01). Treatment with CHF6297 at 30 μg/kg was inactive (2.78 ± 0.28 × 10^5^ cells/mL), whereas the high dose significantly decreased airway neutrophilia compared to the vehicle treated with IAV- and HDM-challenged mice (1.29 ± 0.119 × 10^5^ cells/mL, HDM + IAV = 2.23 ± 0.22 × 10^5^ cells/mL, ^##^ *p < 0.01*). It is of note that the number of neutrophils in the CHF6297-treated group was similar to the number of neutrophils in the BALF of animals treated with HDM only, suggesting that the exacerbation-induced inflammatory response has been completely inhibited by CHF6297. As expected, inhaled budesonide, 100 μg/kg, was inactive in animals showing the exacerbation event (2.08 ± 0.23 × 10^5^ cells/mL). Surprisingly, when budesonide was combined with CHF6297, at 30 μg/kg, a significant and at least additive anti-inflammatory effect was observed (1.21 ± 0.13 × 10^5^ cells/mL, ^##^ *p < 0.01*), while CHF6297 at 100 μg/kg showed a slight increase in neutrophil recruitment inhibition (0.98 ± 0.1 × 10^5^ cells/mL, ^##^ *p < 0.01*).

### Inhibition of i.v. LPS induced TNF-α release in rat blood

**FIGURE 7:**
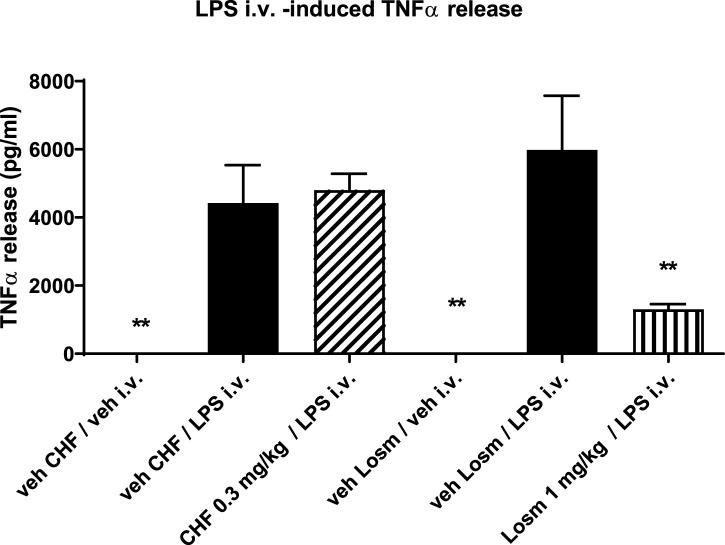
Effect of CHF6297 when dosed i.t. as a micronized dry powder (in the lactose vehicle) on the TNF-α response in the blood after an i.v. LPS challenge. Following the same experimental conditions, losmapimod administered to rats by oral gavage was used as the reference compound. Values are represented as the mean ± SEM (*n* = 9–10 per treatment group); **p < 0.01 vs. LPS i.v. group. In vehicle-treated animals, TNFα in blood was undetectable. Statistical analysis was performed by one-way analysis of variance, followed by Dunnett’s test for multiple comparisons.

To evaluate the systemic activity of CHF6297 upon inhalation delivery, an *in vivo* acute model of LPS-induced inflammation was used. An i.v. dose of LPS (0.3 mg/kg) in rats induces an increase in TNF-α levels in the blood. CHF6297 was administered to the experimental animals using the nose-only tower as a lactose-blended dry powder 2 h prior to LPS challenge. As can be seen in Figure 7, there was no significant inhibition of TNF-α levels with CHF6297 compared to the vehicle (*p* > 0.05). On the other hand, the oral p38 inhibitor losmapimod, with its systemic exposure, induced a marked reduction in TNF-α levels in rat blood. This implies that CHF6297 administered at a dose (0.3 mg/kg) pharmacologically active in the lung via inhalation did not reach pharmacologically effective levels in blood.

### Inhalation pharmacokinetics in rats

**FIGURE 8:**
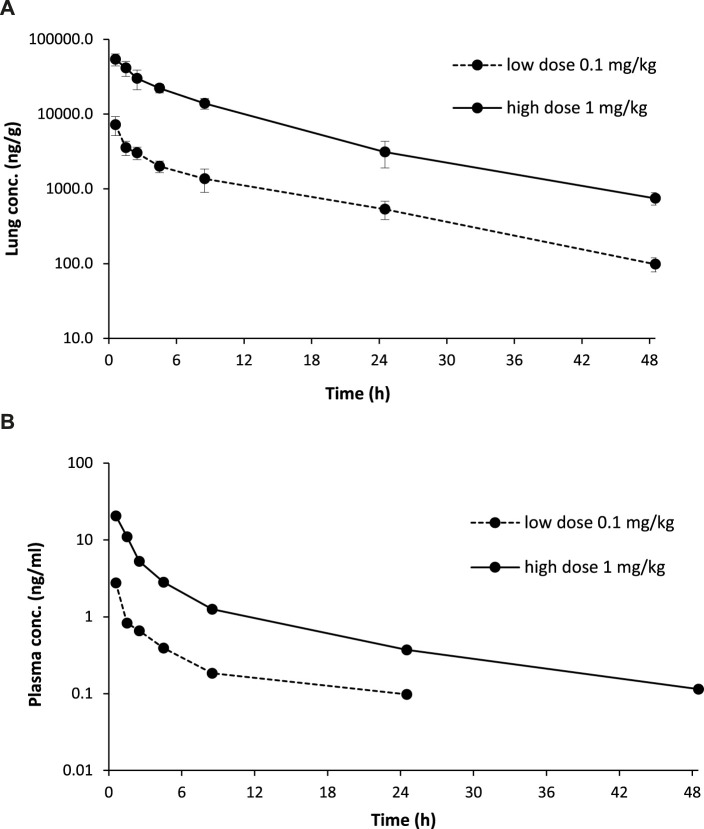
Rat pharmacokinetics (PK) profile of CHF6297. Lung **(A)** and plasma **(B)** PK profiles of CHF6297 after inhalation administration as blend 20% w/w at 0.1 mg/kg (dotted line) and 1 mg/kg (continuous line) to male Wistar rats using the nose-only inhalation delivery system.

After 30 min-inhalation exposure at 2 dose levels (0.1 and 1 mg/kg) using the nose-only inhalation delivery system, CHF6297 showed a PK profile suitable for inhalation administration. Both lung and plasma exposure increased in a proportional manner with the dose. The lung half-life was 10 h at both dose levels. The lung/plasma ratio, calculated by dividing the AUClast in the lung by the AUClast in plasma was approximately 6,000-fold at the two dose levels. The Cmax was reached at the end of the inhalation period in the lung and plasma. The calculated PK parameters for two doses of CHF6297 after inhalation administration in male Wistar rats are reported in Table 2, and the lung and plasma PK profiles are graphically represented in Figure 8.

**TABLE 2:** Lung and plasma PK parameters measured after 30-min administration by passive inhalation of CHF6297 (0.01 and 0.1 mg/kg) to male Wistar rats using the nose-only inhalation delivery system. IAD: immediately after dosing (i.e., 5 min after the end of exposure).

PK parameter	0.1 mg/kg	1.0 mg/kg
Lung Cmax (ng/g)	7,187	54,000
Lung Tmax (h)	IAD	IAD
Lung AUClast (h*ng/g)	42,097	3,72367
Lung t_1/2_ (h)	10.2	10.2
Plasma Cmax (ng/mL)	2.77	20.6
Plasma Tmax (h)	IAD	IAD
Plasma AUClast (h*ng/mL)	7.32	60.1

## Discussion

The p38 MAPKs, in particular the p38α isoform, regulate the production of numerous inflammatory mediators in the lungs of patients affected by chronic and acute inflammatory conditions (Gaffey et al., 2013). Thus, considerable effort has been focused on developing selective p38α inhibitors for the treatment of inflammatory pulmonary diseases. Liabilities for oral p38 inhibitors have been associated with dose-limiting systemic toxicities such as increased liver enzymes and skin rashes, thus limiting their use in chronic respiratory diseases (Chopra et al., 2008). To circumvent such limitations, we developed novel inhaled p38α inhibitors to drive local anti-inflammatory effects in the lungs while minimizing systemic exposure (Armani et al., 2022). In the present study, we thoroughly describe the pharmacological and pharmacokinetic characterization of the drug candidate CHF6297, previously coded as compound 4e (Armani et al., 2022), a novel p38α inhibitor optimized for inhaled delivery as a dry powder through a rational drug design and screening program. CHF6297 inhibitory potency and selectivity were evaluated in a panel of p38 enzymatic assays and in cell-based assays, including PBMCs and airway smooth muscle cells (ASMCs). CHF6297 inhibitory potency on p38α inhibitory activity is in the sub-nanomolar range, with more than 6,000-fold selectivity vs. p38γ and p38δ and no selectivity *versus* p38β, as previously reported for other p38α inhibitors (Norman, 2015; Patel et al., 2018). The p38α rat isoform is also inhibited at the same potency as that of the human isoform. CHF6297 anti-inflammatory potency was compared head-to-head with that of the oral p38 inhibitor losmapimod (Pascoe et al., 2017), and the inhaled p38 inhibitor AZD7624 in cultured human PBMCs stimulated with a bacterial endotoxin and against losmapimod in ASMCs stimulated with cigarette smoke and TNF-α. CHF6297 showed a low nanomolar potency in inhibiting TNF-α in PBMCs and IL-8 release in ASMCs, a potency superior to that of losmapimod and comparable with the reported potency of AZD7624 (Patel et al., 2018). Neutrophilic-dominant pulmonary inflammation is an important feature of COPD. CHF6297, in a dry powder formulation blended with lactose, proved to have suitable lung retention after inhalation delivery in rats and was very effective in reducing LPS-induced neutrophil influx in rat BALF, confirming the rationale for inhibiting p38α in inflamed neutrophilic airways. The robust anti-inflammatory effect of CHF6297 was also confirmed in another model of acute pulmonary inflammation induced by the instillation of IL-1β, a proinflammatory cytokine associated with inflammasome activation and elevated during acute COPD exacerbations (Kubysheva et al., 2022), as well as during the hyperinflammatory response occurring in severe acute respiratory syndrome driven by SARS-CoV-2 (Gilroy et al., 2021). Airway inflammation in COPD, especially during acute exacerbations, is partially insensitive/resistant to inhaled corticosteroids (ICS), thus limiting the efficacy of current ICS-based therapies (Barnes, 2013). We demonstrated that CHF6297 is effective in a corticosteroid-resistant mouse model of allergen-driven pulmonary inflammation exacerbated by viral challenge. In addition, CHF6297 is capable of counteracting neutrophilic influx into the airways resulting from tobacco smoke exposure in mice, another known model of corticosteroid-resistant pulmonary neutrophilic inflammation. Taken together, our data suggest that CHF6297 has the potential to effectively counteract acute pulmonary inflammation including corticosteroid-resistant neutrophilic inflammation. Whether the acute pharmacological effects described in this study will translate into beneficial clinical effects in stable and/or exacerbating COPD and/or severe neutrophilic asthma upon chronic or sub-chronic administration is to be proven. With regard to COPD, there is a need for better knowledge of the pathology drivers to allow careful patient stratification when evaluating compounds in a clinical setting. Although p38α is upregulated in the lungs of COPD patients, the inhaled p38α inhibitor AZD7624 failed to reduce exacerbations in COPD patients in a large phase 2 clinical study (Patel et al., 2018). It does not appear that this was the result of insufficient target engagement in the airways as AZD7624 significantly counteracted the increase from baseline in sputum neutrophils and TNF-α in an LPS challenge study in healthy volunteers (Pehrson et al., 2018). It cannot be excluded that prolonged p38α inhibition in stable COPD patients might not be beneficial due to the complexity of inflammatory pathways underlying COPD and the heterogeneity of such patients. This is possibly a result of “inflammatory escape” during chronic dosing (Chung, 2011), as evidenced by positive effects on inflammatory markers decreasing over the course of a few weeks despite continued dosing (de Buck et al., 2015; Gross, 2016). Therefore, p38α inhibitors, such as CHF6297, might be best positioned in the short-term treatment of inflammation-driven acute exacerbations of disease. Indeed, a potent and selective, oral p38α inhibitor, acumapimod, is under investigation for treatment of acute exacerbations of COPD (AECOPD) (Strâmbu et al., 2019). Moreover, it has been recently proposed acute short-term inhibition of p38α for adjunct treatment of COVID-19, as many of the pathogenic processes that have been thus far associated with disease severity and death, including cytokine storm, clotting, edema, and pulmonary inflammation, are driven by this signaling pathway (Gilroy et al., 2021).

One limitation of this study is the lack of data on CHF6297 in experimental female animals, given also gender differences potentially associated with chronic inflammatory lung diseases. However, such an investigation requires dedicated studies that are beyond the scope of this paper.

In summary, given its excellent pharmacological potency and efficacy as an inhaled anti-inflammatory agent that can be delivered as a dry powder formulation, CHF6297 holds promise as a novel treatment for chronic and acute pulmonary inflammatory conditions such as COPD and severe asthma, as well as AECOPD and COVID-19. Given its satisfactory safety and efficacy profile in preclinical models, CHF6297 has been progressed to a clinical study to investigate the safety, tolerability, pharmacokinetics, and pharmacodynamics of single and repeated doses of CHF6297 in healthy subjects and in patients with COPD (NCT02815488, www.clinicaltrials.gov).

## Data Availability

The original contributions presented in the study are included in the article/Supplementary Material; further enquiries can be directed to the corresponding author.
