# Corrigendum: CHF6297: a novel potent and selective p38 MAPK inhibitor with robust anti-inflammatory activity and suitable for inhaled pulmonary administration as dry powder

**DOI:** 10.3389/fphar.2024.1497520

**Published:** 2024-10-25

**Authors:** Cataldo Martucci, Andrew Dennis Allen, Nadia Moretto, Valentina Bagnacani, Alessandro Fioni, Riccardo Patacchini, Maurizio Civelli, Gino Villetti, Fabrizio Facchinetti

**Affiliations:** Corporate Pre-Clinical R&D, Chiesi Farmaceutici S.p.A., Parma, Italy

**Keywords:** inflammation, P38 alpha, chronic obstructive pulmonary disease, cytokines, neutrophilia

In the published article, there was an error in Figure 1 as published. The chemical structures described in Figure 1 were inaccurate. The corrected Figure 1 and its caption appear below.

**FIGURE 1:**
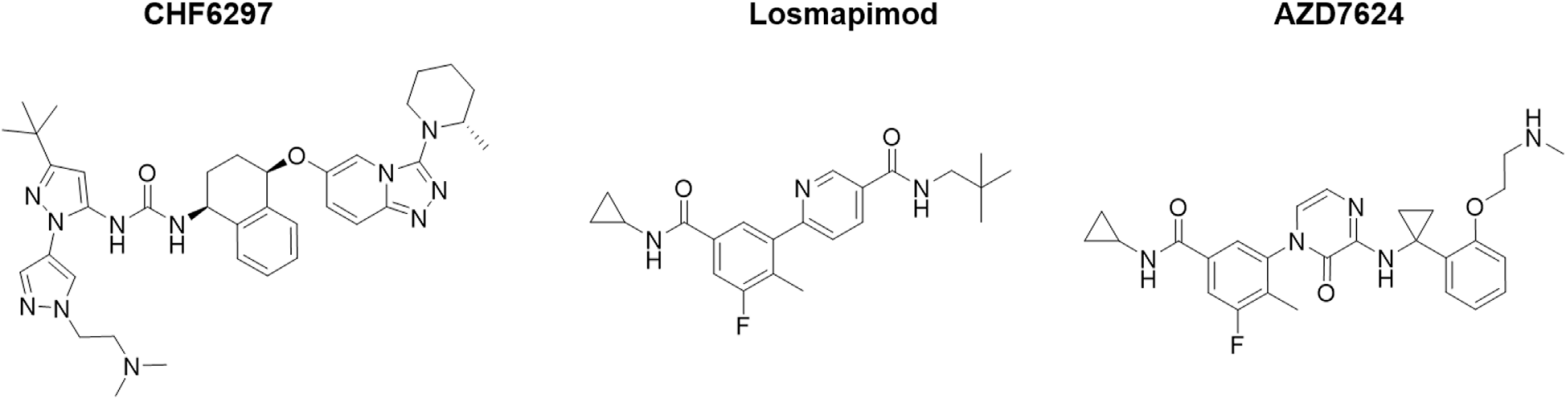
Chemical structures of the p38α inhibitors CHF6297, losmapimod, and AZD7624.

The authors apologize for this error and state that this does not change the scientific conclusions of the article in any way. The original article has been updated.

