# Supplementary material for: CHF6297: a novel potent and selective p38 MAPK inhibitor with robust anti-inflammatory activity and suitable for inhaled pulmonary administration as dry powder
Source: Front Pharmacol. 2024 Mar 14;15:1343941. doi: 10.3389/fphar.2024.1343941 (PMC10973839; doi:10.3389/fphar.2024.1343941)
Supplement: Supplementary file 1 [file Table1.DOCX]

**CHF6297: A Novel Potent and Selective p38 MAPK Inhibitor with Robust Anti-Inflammatory Activity and Suitable for Inhaled Pulmonary Administration as Dry Powder**

Cataldo Martucci, Andrew Dennis Allen, Nadia Moretto, Valentina Bagnacani, Alessandro Fioni, Riccardo Patacchini, Maurizio Civelli, Gino Villetti, and Fabrizio Facchinetti.

Supplementary information

| Target Dose (mg/kg) | Estimated Inhaled Dose | % of Target |
| --- | --- | --- |
| 0.03 | 0.036 | 121 |
| 0.1 | 0.135 | 135 |
| 1.0 | 1.452 | 145 |

**Table 1:** the data in the table show the estimated inhaled dose of CHF6297 dry powder delivered to rat by snout only tower 24 h hours before the LPS challenge. The target doses of 0.03, 0.1 and 1mg/kg are similar to the estimated inhaled dose 0.036, 0.135 and 1.452, demonstrating robust experimental reproducibility. The inhaled dose was derived using the following equation (Alexander et al., 2008):

Delivered dose (μg/kg) =$\frac{Conc \times RMV \times D}{BW}$

Where: Conc. = Concentration of CHF-6297 in air inhaled (µg/L)

RMV = Volume of air inhaled in one minute (L/minute) 0.608 x BW (kg)^0.852^

D = Duration of exposure to the aerosol (minutes)

BW = Body weight (kg)

| CHF6297 nominal dose (mg/kg) | MMAD (µm) | CHF6297 achieved dose (mg/kg) |
| --- | --- | --- |
| 0.1 | 1.4 | 0.101 |
| 1.0 | 1.7 | 1.05 |

**Table 2:** the data in the table show the mass median aerodynamic diameter (MMAD, µm) and the achieved dose of CHF6297 delivered by nose-only inhalation delivery system at the doses of 0.1 and 1 mg/kg during the PK study.

**Fig 1.**

**Inhalation Tower System**
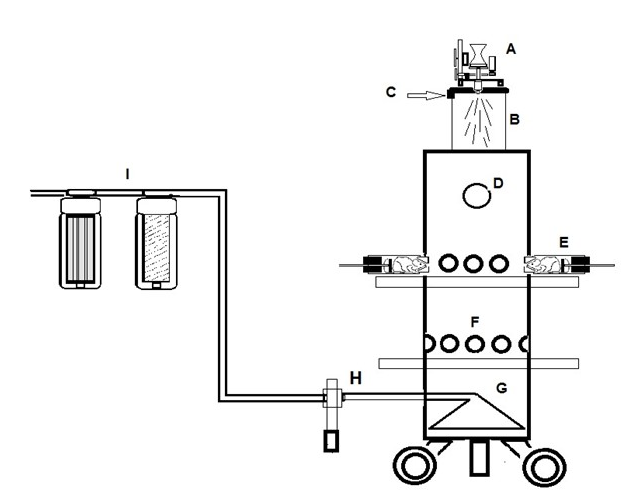


**Figure 1.** A schematic depiction of the inhalation towers utilized in this study with the description of its main components. A. Wrights Dust Feed. B. Pre-Chamber. C. Tangential air inlet. D. Viewing port in top section. E. Restraint tube attached to the tower. F. Vacant animal ports closed off with bungs. G. Conical exhaust plenum. H. Aerosol Monitoring device. I. Filtration units in extract.
